# A Comparison of Methods for Identifying Enterobacterales Isolates from Fish and Prawns

**DOI:** 10.3390/pathogens11040410

**Published:** 2022-03-28

**Authors:** Arkadiusz Józef Zakrzewski, Urszula Zarzecka, Wioleta Chajęcka-Wierzchowska, Anna Zadernowska

**Affiliations:** Department of Industrial and Food Microbiology, University of Warmia and Mazury, Plac Cieszyński 1, 10-726 Olsztyn, Poland; urszula.zarzecka@uwm.edu.pl (U.Z.); wioleta.chajecka@uwm.edu.pl (W.C.-W.); anna.zadernowska@uwm.edu.pl (A.Z.)

**Keywords:** *16S rRNA* sequencing, Enterobacterales, Enteropathogens, EnteroTest 24N, MALDI-TOF MS

## Abstract

Enterobacterales is a prevalent order, which inhabits a variety of environments including food. Due to the high similarities between pathogenic and non-pathogenic species, their identification might be difficult and laborious, and therefore there is a need for rapid and precise identification. The aim of this study was to compare the effectiveness of the available methods of identifying order *Enterobacterales* strains isolated from fresh fish and shrimps (n = 62). The following methods were used in this study: biochemical, sequencing and identification using the matrix-assisted laser desorption/ionization time-of-flight mass spectrometry (MALDI-TOF MS). For this purpose, biochemical identification was performed with the use of the EnteroTest 24N set, while the identification using the MALDI-TOF MS technology was operated on VITEK^®^ MS. Results were compared with identification made by 16S rRNA sequencing. The results of the study showed that conventional identification methods might provide a false result. Identification by VITEK^®^ MS to the species level was correct at 70.97%, and the accuracy of EnteroTest 24N identification did not exceed 50.0%. The genus identification reached 90.32% for the MALDI-TOF technique, while for EnteroTest 24N it was nearly 70.0%. Due to errors in identification, especially of pathogenic organisms, the use of each of these methods should be confirmed by another method, preferably sequencing.

## 1. Introduction

Order Enterobacterales is a group of Gram-negative, facultatively anaerobic, non-spore-forming, rod-shaped microorganisms. These microorganisms are widespread in the environment, although many species constitute the harmless commensals of animals. However, Enterobacterales are able to live in various ecological niches such as soil, water and food, especially of animal origin. Many species of Enterobacterales are important human and animal pathogens, and few are considered pathogenic to plants and insects. The most important pathogenic species for humans and livestock are *Salmonella* spp., pathogenic *Escherichia coli*, including Enteroinvasive *E. coli*, Enterotoxigenic *E. coli*, Enteroaggregative *E. coli* and others, *Yersinia enterocolitica*, *Shigella* spp. and *Cronobacter* spp. Other members of the family are regarded as opportunistic pathogens, especially in clinical settings, including *Klebsiella* spp., *Serratia* spp., *Hafnia* spp. and *Citrobacter* spp. Due to the great similarity between individual species, the identification of Enterobacterales is often challenging [1]. Various methods of microorganisms’ identification are known. Conventional microbiological techniques, based on morphological and biochemical characterizations, are the most frequently used for bacterial species’ identification. However, the results of those methods might be unequivocal. Genome-based identification is expected to be performed in order to confirm representatives of the Enterobacterales [2].

The use of tests based on biochemical methods, focusing on glycerol utilization, sugar fermentation or the presence of enzymes, for the identification of Enterobacterales, has been the standard for many years. Many commercial tests are now available for use. While some studies have shown high accuracy rates associated with biochemical tests, other studies have reported less satisfactory identification [3]. Additionally, biochemical methods may not be reliable for environmental isolates because of small biochemical differences in adequate information on environmental bacteria in databases. In addition, commercially available identification systems are sometimes unable to identify some organisms [4,5]. Commercial identification systems such as the analytical profile index (API, bioMérieux) or equivalent are most frequently used for this purpose; however, resource restrictions limit their application [6].

Matrix-assisted laser desorption/ionization time-of-flight mass spectrometry (MALDI-TOF MS) has been implemented as a cost- and time-effective alternative to *16S rRNA* gene sequencing. The basic assumption of this method is made due to the fact that each species of bacteria has a characteristic protein expression ‘fingerprint’. In this method, mass signals from bacterial proteins are detected and their unique mass spectra are determined. The particles’ time of flight to the detector depends on the mass-to-charge ratio. Spectra are compared with reference bacterial strains in a mass spectra library, which is able to differentiate the bacteria to their genus, species or sub-group levels [4,7,8]. A major advantage of MALDI-TOF MS is the rapid time used in accurate identification of the organism compared with conventional methods and the small amount of biological material required [6].

Genotypic identification has been developed as an alternative or a complement to phenotypic methods. Genetic-based methods, mainly sequencing, are often employed to identify and classify representative bacterial isolates. The key advantage of gene sequencing is its applicability across whole bacterial and archaeal domains. The most discriminative are *gyrB* 16S rRNA genes. The *gyrB* gene has previously been used for describing phylogenetic relationships between Enterobacterales [9]. It has been considered more discriminating than 16S rRNA, since it allows the differentiation and identification of closely related species within the order Enterobacterales [4]. Using sequencing, numerous bacterial genera and species have been reclassified and renamed, and classification of uncultivable bacteria has been made possible [10]. Although molecular approaches have been proven to be very sensitive, they have their own limitations. Molecular approaches are limited mainly by the extent of their databases, and often require complex procedures, trained personnel and expensive, specialized equipment [11].

The aim of this study was to compare the effectiveness of the available methods to identify strains of the order Enterobacterales isolated from fresh fish and shrimps. Identification was performed using the EnteroTest 24N and the MALDI-TOF technique, in comparison to 16S rRNA sequencing.

## 2. Results

Identification of reference strains using the MALDI-TOF technique was 100% effective. All ten strains have been identified for the species level with a confidence level of 99.8%. Whereas six out of ten strain identifications by EnteroTest 24N tests were identified at a good to excellent level. Identification of the strain *Salmonella enterica* subsp. *enterica* (ATCC^®^ BAA-664™) at an excellent level afforded a positive, more detailed result because the analysis showed that the strain was *Salmonella* serovar Enteritidis. In the case of three strains, the identification analysis at the species and genus levels was correct at a given level. However, in one case, the EnteroTest 24N provided incorrect identification: the reference strain *Salmonella enterica* subsp. *enterica* (ATCC^®^ BAA-664™) was identified as *Salmonella* serovar Enteritidis. Nevertheless, information was obtained that the identification was at the excellent level. The results are presented in Table 1.

Among all the tested strains, 62 morphotypes were selected for further testing. Statistical analysis showed that MALDI-TOF performed better than EnteroTest 24N, both in terms of genus (*p* = 0.0005) and species identification (*p* = 0.00001). The species identification sensitivity by MALDI-TOF MS yielded 83.33%, while for EnteroTest 24N, it was 100%. Due to misidentification, each strain of the genus *Aeromonas* spp. SP, PPV and NPV reached 0 for EnteroTest 24N (Table 2). Additionally, EnteroTest 24N was characterized by a very low AC (37.68%), while MALDI-TOF was characterized by a higher correctness, at the level of 69.56%.

**Figure 1:**
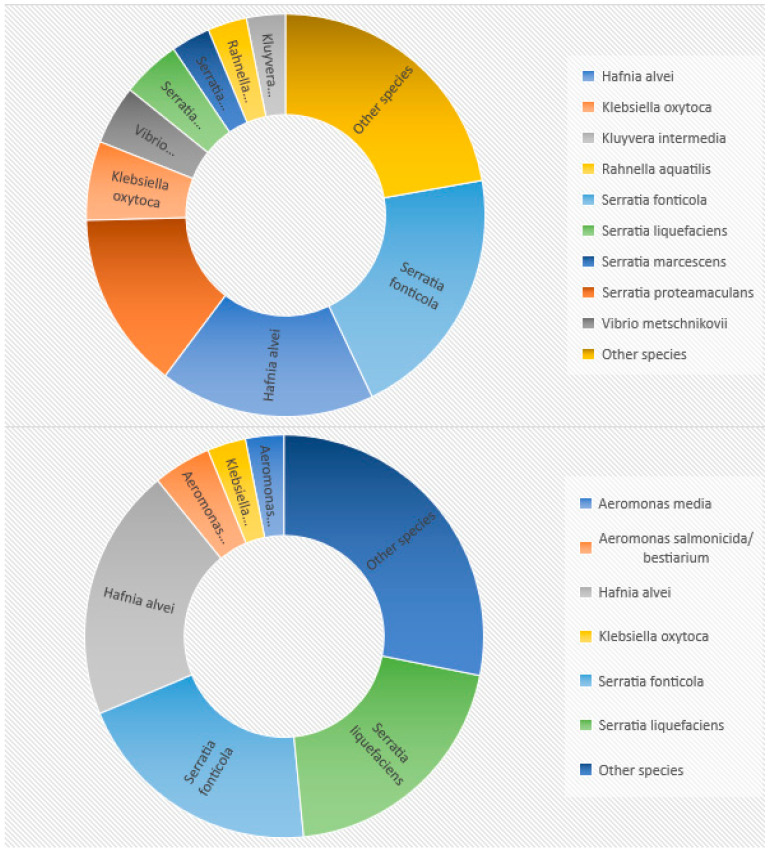
Percentage distribution of species identified using (**top**) EnteroTest 24N and (**bottom**) MALDI-TOF.

On the basis of the EnteroTest 24N analyses, it was found that the most isolated strains belonged to *Serratia fonticola* species (20.31%). Less frequent strains were *Hafnia alvei* (17.19%), *Serratia proteamaculans* (14.06%), *Klebsiella oxytoca* (6.25%) and *Serratia liquefaciens* (4.69%). In the case of MALDI-TOF, the identification results differed from EnteroTest 24N. Three strains were identified equally as often: *Hafnia alvei*, *Serratia fonticola* and *Serratia liquefaciens*. The percentage of each of them was 20.31%. The remaining species were represented by a meager number of strains: *Aeromonas salmonicida/bestiarium* (4.69%), *Aeromonas media* and *Klebsiella oxytoca* (3.125% each). The results of the percentage distribution are shown in Figure 1.

**Figure 2:**
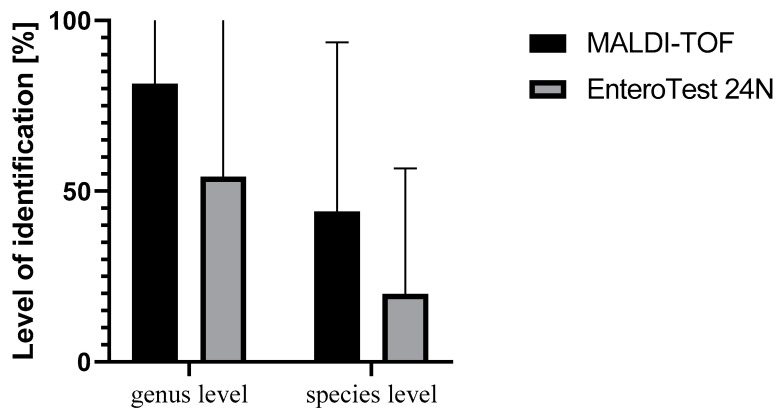
Percentage results of correct identification obtained from EnteroTest 24N and MALDI-TOF methods in comparison with the sequencing method.

The comparison of the tested methods—EnteroTest 24N and MALDI-TOF—with the sequencing method as a reference showed that 70.97% of the strains were identified to the species level with the MALDI-TOF technique, compared with 41.9% using the EnteroTest 24N. In the case of identification to the genus level, the MALDI-TOF technique indicated correct identification at the level of 90.32%, while in the case of EnteroTest 24N it was nearly 70.0% (Figure 2, Supplementary Table S1). Neither method was able to identify both species and genus for *Citrobacter koseri, Enterobacter kobei, Enterobacter ludwigii* and *Lelliottia nimipressuralis*. For six strains in each test, the identification was correct only to the genus level, using MALDI-TOF: *Aeromonas rivipollensis, Aeromonas hydrophila, Klebsiella variicola, Klebsiella pneumoniae, Leclercia adecarboxylata* and *Serratia proteamaculans,* and using the EnteroTest 24N test: *Klebsiella oxytoca, Klebsiella variicola, Klebsiella pneumoniae, Serratia proteamaculans, Serratia liquefaciens* and *Yersinia enterocolitica*. Correct identification for both methods was observed for only two strains of the order Enterobacterales: *Enterobacter cloacae* and *Rahnella aquatilis.* The results are shown in Table 3.

## 3. Discussion

Rapid detection and identification of microorganisms is essential in the food industry. The main challenge of microorganisms’ identification using traditional methods from the food industry is their high variability, also characteristic for environmental tests [12,13].

Identification by biochemical tests is a conventional method, although it is still routinely used to identify certain pathogens. Due to the high preparation costs, time-consuming procedures and prolonged results, conventional methods are rarely used. Currently, automatic and semi-automatic systems are the most commonly used biochemical methods for identifying microorganisms. The most commonly used automated techniques are VITEK^®^ 2 Compact (bioMerieux, Marcy l’Etoile, France) and BD Phoenix (BD Diagnostics, Franklin Lakes, NJ, USA) [14].

The market offers numerous semi-automatic biochemical tests for the identification of Gram-negative bacteria, including MICROBACT™ (Thermofisher), The Microgen ™ (Microbiology Interational), Microgen Biochemical Identification Kits (Microgen Bioproducts), EnteroTest 24N (Erba-Lachema, Brno, Czech Republic) and API^®^ (bioMerieux, Marcy l’Etoile, France), of which the API^®^ system is the most popular test [14].

Although there are many studies using EnteroTest 24N (Erba-Lachema, Brno, Czech Republic) to identify microorganisms [15,16,17,18], no research has been carried out to verify their accuracy before. This test differs significantly from the common API 20E semi-automatic system and the carts for the VITEK^®^ 2 compact automatic system (bioMérieux, Marcy l’Etoile, France). EnteroTest 24N contains adonitol, cellobiose, trehalose and dulcitol as fermentation substrates, while API 20E contains amylogdalin and arabinose. The EnteroTest 24N test does not include oxidase, gelatinase and tryptophan deamination test substrates. Although API systems are used worldwide, research on their accuracy was conducted mainly in the 1970s and 1980s, and therefore they will not be included in the discussion [19].

Our research has shown that 80% of the reference strains were correctly identified to the species level and 100% to the genus level using the EnteroTest 24N test, while for MALDI-TOF, the accuracy was 100%. In the case of environmental strains, identification using the MALDI-TOF technique was much more effective than EnteroTest 24N tests and reached 70.97% of correctly identified strains, compared to 41.97% obtained by EnteroTest 24N.

In a study comparing different methods of identifying psychrotropic bacteria isolated from raw milk by Nuwan et al., it was found that Gram-negative rods were identified by the API system at 60.5% to the strain level and 97.3% to the genus level. In the same study, the MALDI-TOF technique was used and an identification of 73.7% to the genus level and 63.2% to the strain level was obtained [20].

A study by Richter et al. comparing the specificity of the bioMerieux VITEK^®^ MS system in the identification of *Enterobacteriaceae* found that the identification was consistent with the reference identification method for 96.7% of the tested isolates, with 83.8% consistent with the species level and 12.8% limited to identification at the genus level. Another research team comparing identifications between the two MALDI-TOF systems showed that VITEK^®^ MS correctly identified *Enterobacteriaceae* at the strain and genus level for 94.1% and 92.2%, respectively [21].

Among the pathogenic species belonging to the order Enterobacterales, the VITEK^®^ MS system correctly identified *Yersinia enterocolitica*, while the biochemical test correctly identified strains only at the genus level, specifying the non-pathogenic species *Yersinia frederiksenii.* Comparing the possibility of identifying *Yersinia enterocolitica* isolated from minced meat showed that API 20E is the most suitable for research on this pathogen [22]. The same conclusions were reached by Roger et al. in their study of *Yersinia enterocolitica* and *Yersinia pestis* strains isolated from various sources, including food, using the MALDI-TOF technique [23,24].

In the case of the identification of *Citrobacter korseri*, responsible primarily for urinary tract infections [25], it has been shown that none of the methods tested identified the species either to the species or genus level. Kolínská et al. obtained quite different results, obtaining 100% correctness in the study of *C. korseri* strains both by EnteroTest 24N tests and identification using the MALDI-TOF technique. The authors obtained differences in identification for strains from species *C. youngae*, *C. braakii* and *C. gillenii* [26].

Considered the most pathogenic species of the genus *Enterobacter*, *E. cloacae* was correctly identified using both EnteroTest 24N and VITEK^®^ MS. Pavlovic et al. found that the specificity of the MALDI system was insufficient to distinguish *Enterobacter asburiae*, *Enterobacter hormaechei*, *Enterobacter kobei* and *Enterobacter ludwigii* from *Enterobacter cloacae*. As many as 11 out of 56 (20%) isolates from the *E. cloacae* group could not be clearly identified as a specific species using MALDI-TOF MS [27].

Worrying identification results were obtained in the genus *Klebsiella* because the pathogenic *Klebsiella pneumioniae* was identified only to the genus level for both methods, while the pathogenic *Klebsiella oxytoca* was also correctly identified by the MALDI-TOF technique, whereas the EnteroTest 24N identification reached only 50%. Research shows that the gold standard for this pathogen is not a successful method and *K. pneumoniae* was identified correctly in only 32.9% [28]. In the study of 76 strains belonging to the genus *Klebsiella*, all of them were correctly identified to the species *K. oxytoca* and *K. pneumoniae* using the VITEK^®^ MS system and the VITEK^®^ 2 automatic system [29].

In many descriptions of the MALDI-TOF MS technique, one of the advantages is the low cost of a single identification, which is USD 0.5–1.75, whereas for the gold standard API, the price is about USD 9.95. However, the cost of the device is important. In the case of EnteroTest 24N, basic laboratory tools or software for easier and faster interpretation of the results are sufficient, however, it is not necessary. For MALDI-TOF MS, this costs around USD 150,000–250,000, which many laboratories cannot afford [30].

## 4. Materials and Methods

### 4.1. Sample Collection and Strain Isolation

In the study, 62 strains from the collection of the Department of Industrial and Food Microbiology at the University of Warmia and Mazury in Olsztyn were used. Strains were isolated from 117 samples of raw fish meat and 39 raw shrimps, including 58 samples of Atlantic salmon (*Salmo salar*), 59 samples of rainbow trout (*Oncorhynchus mykiss*) and 39 samples of prawns (*Penaeus monodon*). Strains were grown from frozen stocks, kept in the Microbank (Biomaxima, Lublin, Poland) at −80 °C in 5 mL of Brain Heart Infusion broth (Merck, Darmstadt, Germany) overnight at 37 °C.

Isolation of Enterobacterales was performed by putting together 10 g of raw fish with 90 mL of saline, then a series of 10-fold dilutions were performed in agreement with standard methods for initial suspension and decimal dilutions of test samples for microbiological examination (ISO 6887-1:2017-05) [31]. A total of 0.1 mL of the dilution was inoculated onto violet red bile glucose (VRBG) agar and incubated for 24 h at 37 °C. In order to confirm that typical colonies belong to the order Enterobacterales, an oxidase test was performed. Additionally, 10 strains from the American Type Culture Collection (ATCC) were identified as reference strains.

### 4.2. EnteroTest 24N

Identification of the isolated strains was carried out using EnteroTest 24N (Erba-Lachema, Brno, Czech Republic). The test provides fermentation of adonitol, cellobiose, dulcitol, inositol, d-mannitol, melobiose, raffinose, l-rhamnose, d-sorbitol, sucrose and trehalose, malonatehydrolisis of esculine, production of acetoin, ß-galactosidase and hydrogen sulfide, indole deamination of phenylalanine, lysine, arginine and ornithine and utilization of citrate. The test was carried out by following the manufacturer’s instructions. Briefly, the suspension was prepared from a 24 h culture on Trypticase Soy Broth (TSB) (Merck, Darmstadt, Germany) in a 0.9% saline solution to obtain turbidity equal to the McFarland 1.0 turbidity. The results were obtained after 24 h of incubation at 37 °C. The commercial ErbaExpert software (Erba-Lachema, Brno, Czech Republic) was used for identification of the strain of Gram-negative fermentative oxidase-negative rods. The confidence level was determined at one of the following levels: excellent, very good, good, species, genus, low and non-identification.

### 4.3. MALDI-TOF Identification

Measurements were performed using VITEK^®^ MS (bioMérieux, Marcy l’Etoile, France), with an acceleration voltage of 200 kV, mass range of 2–20 kDa, laser frequency of 50 Hz and an extraction delay time of 200 ns. All mass fingerprints were analyzed by the VITEK^®^ MS v2.0 MALDI-TOF mass spectrometry systemV2.0, research use only (RUO; SARAMIS version 4.13) databases (bioMérieux, Marcy l’Etoile, France).

Isolates were tested in duplicate using the direct transfer protocol according to the manufacturers’ recommendations. Briefly, the isolates were cultured for 48 h at 30 °C on TSA (Merck, Darmstadt, Germany), and were then transferred to the target plate. One microliter of MALDI matrix VitekMS-CHCA (bioMérieux, Marcy l’Etoile, France) was added to the spots. After crystallization of the matrix solution, the target was loaded into the MALDI-TOF MS, and the analysis was started. The confidence level was determined in percentage.

### 4.4. Sequencing

In the first step, total DNA was extracted using the Genomic Mini AX Bacteria Spin kit (A&A Biotechnology, Gdańsk, Poland) according to the manufacturer’s instructions. For identification, 16S rRNA sequences of each analyzed strain were used. The positive control was two strains of *E. coli* (*E. coli* ATCC^®^ 8739™ and *E. coli* ATCC^®^ 25922™), and the negative control was PCR-grade water. For amplification, the primer sets 27F: 5′-AGAGTTTGATCCTGGCTCAG-3′ and 1492R 5′-GGTTACCTTGTTACGACTT-3′ were used [32]. The PCR was performed under the following conditions: 3 min initial denaturation at 95 °C, 35 cycles of denaturation (30 s at 95 °C), annealing (30 s at 55 °C), extension (1.5 min at 72 °C) and a final extension at 72 °C for 7 min. Before sequencing, the PCR reaction products were cleaned with Clean-up (A&A Biotechnology, Gdańsk, Poland) according to the manufacturer’s instructions. Sequencing was performed by Genomed Company (Warsaw, Poland). All sequences were compared with the NCBI GenBank database (National Center of Biotechnology Information, Bethesda, MD, USA) using the Basic Local Alignment Search Tool (BLAST). A phylogenetic tree was constructed based on the Neighbor-Joining method with ~1000 base pairs of 16S rRNA sequences, compared to other bacterial strains obtained from the NCBI GenBank database. For alignment and tree construction, MEGA6 software [33] was used. The sequences were deposited in the GenBank database (https://www.ncbi.nlm.nih.gov/genbank (accessed on 16 June 2021), accession Nos. MK404730, MK404731 and MK404732).

### 4.5. Statistical Analysis

The evaluation of the assays was calculated for each method as follows:

Sensitivity (*SE*):

(1) $$SE=\frac{TP}{TP+TN}\times 100\%$$

Specificity (*SP*):

(2) $$SP=\frac{TN}{TN+FP}\times 100\%$$

Positive predictive value (*PPV*):

(3) $$PPV=\frac{TP}{TP+FP}\times 100\%$$

Negative predictive value (*NPV*):

(4) $$NPV=\frac{TN}{FN+TN}\times 100\%$$

Relative accordance correctness (*AC*):

(5) $$AC=\frac{TP+TN}{N}\times 100\%$$

Positive likelihood ratio (*PLR*):

(6) PLR=SE/(1−SP)

Negative likelihood ratio (*NLR*):

(7) NLR=(1−SE)/SP

where: *TP*, true positive; *TN*, true negative; *FP*, false positive; *FN*, false negative.

A false positive result was defined as a correct genus or family level identity belonging to the order Enterobacterales, whereas a false negative result was defined as the lack of identification. Comparison of the VITEK^®^ MS with the EnteroTest 24N for the identification of genus or species was performed using McNemar’s test, a test of paired proportions. *p*-values of 0.05 were considered statistically significant. Statistical analysis was performed using PQStat version 1.8.0 (PQStat softwere, Poznan, Poland) [34].

## 5. Conclusions

The MALDI-TOF technique and EnteroTest 24N system are often used for the diagnosis of food-related microorganisms. Both methods easily identify typical and well-understood strains from the ATCC collection, whereas due to a greater environmental variation, the effectiveness of their identification is lowered. Due mainly to the use of information on ribosomal proteins, MALDI-TOF MS achieved higher reliability of the identification results. On the other hand, the EnteroTest 24N test, due to the high variability of organisms and the limited number of checked features, did not show such a high level of identification. Although the much better results obtained with the MALDI-TOF MS technique and the low cost of a single identification are in favor of using it in routine work, due to errors in identification, especially of pathogenic organisms, the use of each of these methods should be confirmed using a different method, preferably genotyping.

## Figures and Tables

**Table 1:** Comparison of identification of reference strains using MALDI-TOF and EnteroTest 24N.

No.	Strain	Source	MALDI-TOF		EnteroTest 24N	
			ID	Confidence Level	ID	Confidence Level
1	*Escherichia coli* (ATCC^®^ 8739™)	feces	*Escherichia coli*	99.8%	*Escherichia coli*	excellent
2	*Escherichia coli* (ATCC^®^ 25922™)	clinical isolate	*Escherichia coli*	99.8%	*Escherichia coli*	excellent
3	*Hafnia alvei* (ATCC^®^ 51815™)	milk	*Hafnia alvei*	99.8%	*Hafnia alvei*	good
4	*Serratia marcescens* subsp. *marcescens* (ATCC^®^ 13880™)	pond water	*Serratia marcescens*	99.8%	*Serratia marcescens*	very good
5	*Enterobacter cloacae* subsp. *cloacae* (ATCC^®^ BAA-1143™)	ND	*Enterobacter cloacae*	99.8%	*Enterobacter cloacae* subsp. *cloacae*	very good
6	*Klebsiella pneumoniae* subsp. *pneumoniae* (ATCC^®^ 700603™)	clinical isolate	*Klebsiella pneumoniae*	99.8%	*Klebsiella oxytoca*	genus
7	*Salmonella enterica* subsp. *arizonae* (ATCC^®^ 13314™)	ND	*Salmonella enterica* subsp. *arizonae*	99.8%	*Salmonella enterica* subsp. *arizonae*	species
8	*Salmonella enterica* subsp. *enterica* (ATCC^®^ 14028™)	tissue	*Salmonella enterica* subsp. *enterica*	99.8%	*Salmonella* serovar Enteritidis	genus
9	*Salmonella enterica* subsp. *enterica* (ATCC^®^ BAA-664™)	ND	*Salmonella enterica* subsp. *enterica*	99.8%	*Salmonella* serovar Enteritidis	excellent
10	*Salmonella enterica* subsp. *enterica* (ATCC^®^ 7001™)	ND	*Salmonella enterica* subsp. *enterica*	99.8%	*Salmonella enterica* subsp. *arizonae*	species

Abbreviations: ND, not defined.

**Table 2:** MALDI-TOF and EnteroTest 24N characteristics.

Method	SE (%)	SP (%)	PPV (%)	NPV (%)	AC (%)	PLR	NLR
MALDI-TOF MS	83.33	30.76	68.96	72.72	69.56	1.20	0.54
EnteroTest 24N	100	0	0	0	37.68	1	-

SE: Sensitivity, SP: Specificity, PPV: Positive predictive value, NPV: Negative predictive value, AC: Relative accordance correctness, PLR: Positive likelihood ratio, NLR: Negative likelihood ratio.

**Table 3:** Identification of strains isolated from raw fish and shrimps by the VITEK MS v2.0 MALDI-TOF mass spectrometry system and EnteroTest 24N compared to 16S rRNA sequencing.

Family:	Genus:	Species (Identified by 16S RNA Sequencing)	No. of Isolates	MALDI-TOF Identification (%)		EnteroTest 24N (%)	
				Genus	Species	Genus	Species
*Aeromonadaceae*	*Aeromonas*	*A. salmonicida*	4	75.00	75.00	0.00	0.00
		*A. rivipollensis*	1	100.00	0.00	0.00	0.00
		*A. hydrophila*	1	100.00	0.00	0.00	0.00
*Enterobcteriaceae*	*Buttiauxella*	*B. agrestis*	1	100.00	100.00	0.00	0.00
	*Citrobacter*	*C. koseri*	1	0.00	0.00	0.00	0.00
	*Enterobacter*	*E. cloacae*	1	100.00	100.00	100.00	100.00
		*E. kobei*	2	0.00	0.00	0.00	0.00
		*E. ludwigii*	2	0.00	0.00	0.00	0.00
	*Klebsiella*	*K. oxytoca*	2	100.00	100.00	100.00	50.00
	*Klebsiella*	*K. variicola*	1	100.00	0.00	100.00	0.00
	*Klebsiella*	*K. pneumoniae*	1	100.00	0.00	100.00	0.00
	*Leclercia*	*L. adecarboxylata*	1	100.00	0.00	0.00	0.00
	*Lelliottia*	*L. nimipressuralis*	2	0.00	0.00	0.00	0.00
		*L. amnigena*	1	100.00	100.00	0.00	0.00
	*Raoultella*	*R. ornithinolytica*	1	100.00	100.00	0.00	0.00
*Erwiniaceae*	*Pantoea*	*P. agglomerans*	1	100.00	100.00	0.00	0.00
*Hafniaceae*	*Hafnia*	*H. alvei*	13	92.86	92.86	78.57	78.57
*Yersiniaceae*	*Rahnella*	*R. aquatilis*	1	100.00	100.00	100.00	100.00
	*Serratia*	*S. fonticola*	13	92.30	92.30	92.30	92.30
		*S. proteamaculans*	1	100.00	0.00	100.00	0.00
		*S. liquefaciens*	10	100.00	100.00	100.00	20.20
	*Yersinia*	*Y. enterocolitica*	1	100.00	100.00	100.00	0.00

## Data Availability

Not applicable.

## Supplementary Materials

The following supporting information can be downloaded at: https://www.mdpi.com/article/10.3390/pathogens11040410/s1, Table S1: Identification of the strains used in the study.
